# Phylogeny, Diversification Rate, and Divergence Time of *Agave sensu lato* (Asparagaceae), a Group of Recent Origin in the Process of Diversification

**DOI:** 10.3389/fpls.2020.536135

**Published:** 2020-11-09

**Authors:** Ofelia Jiménez-Barron, Ricardo García-Sandoval, Susana Magallón, Abisaí García-Mendoza, Jorge Nieto-Sotelo, Erika Aguirre-Planter, Luis E. Eguiarte

**Affiliations:** ^1^Laboratorio de Evolución Molecular y Experimental, Departamento de Ecología Evolutiva, Instituto de Ecología, Universidad Nacional Autónoma de México, Ciudad Universitaria, Mexico City, Mexico; ^2^Departamento de Biología, Facultad de Ciencias, Universidad Nacional Autónoma de México, Ciudad Universitaria, Mexico City, Mexico; ^3^Departamento de Botánica, Instituto de Biología, Universidad Nacional Autónoma de México, Ciudad Universitaria, Mexico City, Mexico; ^4^Jardín Botánico, Instituto de Biología, Universidad Nacional Autónoma de México, Ciudad Universitaria, Mexico City, Mexico

**Keywords:** evolutionary radiation, comparative method, extinction, speciation, inflorescence, ancestral state reconstruction, Bayesian inference, ITS

## Abstract

*Agave sensu lato* is one of the most diverse and complex genera of Asparagaceae, with more than 250 species. The morphological, ecological, and evolutionary diversity of the group has complicated its taxonomical study. We conducted phylogenetic analyses of DNA sequence data to reconstruct the phylogenetic relationships of the *Agave* genus. We included 107 species of the Asparagaceae family from which 83 correspond to the *Agave sensu lato* clade (*Agave sensu stricto* + *Polianthes* + *Manfreda* and *Prochnyanthes*, which together represent 30% of the genus) and as outgroups the genera *Dasylirion*, *Hesperoyucca*, *Chlorogalum*, *Camassia*, *Hesperaloe*, *Yucca*, *Beschorneria*, and *Furcraea*, in order to estimate the age and propose the history of their diversification. Previous studies postulated the relevance of the Miocene in the speciation rates of the agaves, as well as the relevance of the type of inflorescence in its diversification. However, these assertions have not been well supported. The analysis of chloroplast regions resulted in low resolution, which could be the consequence of the few variable sites. On the other hand, the internal transcribed spacer (ITS) implemented in our analysis ensued in higher resolution and better support values. Our phylogenetic analyses recovered five groups; one is the Striatae group, which is the sister group to *Agave sensu stricto* clade. Within this clade, we found three main groups with high support; these groups are not related with previous morphological proposals. We also analyzed the dates of origin and diversification rates. A Bayesian analysis of macroevolutionary mixtures indicated two significant shifts; the first was identified at 6.18 Ma, where the speciation rate increased to 4.10 species/Mya, this shift occurred during the late Miocene period, characterized by the emergence of arid biomes in North America. The second was identified at a stem age of 2.68 Ma where the speciation rate increased to 6.04 species/Mya. Concerning the ancestral reconstruction state of the inflorescence type in the *Agave sensu stricto* clade, the spike inflorescence character was predominant in the early-diverging groups, whereas the late-diverging groups present panicle inflorescences as the predominant character and higher speciation rates.

## Introduction

The process of evolutionary radiation has been considered as one of the most important sources of biological diversity, through relatively rapid differentiation from a single ancestor into new species that inhabit a variety of environments and may differ in the characters they use to exploit them (Schluter, 1996, 2000; Olson and Arroyo-Santos, 2009). An evolutionary radiation is a complex process that may involve phenotypic and physiological differentiation, adaptation, speciation, and extinction (Schluter, 2001; Coyne and Orr, 2004; De Queiroz, 2007; Givnish, 2010; Nosil, 2012).

The factors that influence the net species diversification rate–that is to say, the net result of speciation and extinction for each taxon–are multiple and complex (Scott and Arnold, 1995; Magallón and Sanderson, 2001; Magallón and Castillo, 2009; Arakaki et al., 2011; Schlumpberger, 2012; Schlumpberger and Renner, 2012; Van der Niet and Johnson, 2012; Hernández-Hernández et al., 2014). These include extrinsic factors, such as physical space, climate, other organisms, or available habitats, or intrinsic factors, such as morphological or physiological traits, characters that affect the body’s adequacy through its growth, survival, and/or reproduction (Glor, 2010; Losos, 2010; Bouchenak-Khelladi et al, 2015). The comparative study of lineages suspected of having radiation events with those that apparently have not experienced radiation events may help to identify the factors that influenced such changes in their diversification rates.

One of the challenges when studying the diversification of a group is understanding the factors influencing speciation and extinction rates, since the possible factors are many and it is difficult to disentangle which one or if several factors are affecting diversification (Zamora-Abrego et al., 2013). The comparative method allows us to make formal and statistical comparisons between species and thus analyze information, such as morphological, physiological, or ecological characters, by incorporating information on the phylogenetic relationships of the group of interest (Harvey and Pagel, 1991; Garamszegi, 2014). In this way, the comparative method has allowed the evaluation of the extent at which variation of a character is due to its evolutionary history or to adaptive pressures (Morales, 2000; Rezende and Garland, 2003).

Two diversification events have been proposed for the *Agave sensu lato* clade since its origin in the Miocene 7.9–10.2 Ma (Good-Avila et al., 2006; Scheinvar et al., 2017). The first occurred ∼6–8 Ma and correlates with the emergence of arid biomes in Mexico. The second was ∼3–2.5 Ma, and it was apparently associated to the evolution of reproductive characters and pollination syndromes (Eguiarte et al., 2000; Good-Avila et al., 2006; Rocha et al., 2006). The genus *Agave* has experienced constant taxonomic revisions, in part due to their high morphological variation and species diversity (Gentry, 1982; Álvarez de Zayas, 1995; García-Mendoza, 1995, 2002; Hernández-Sandoval, 1995; García-Mendoza et al., 2019) in addition to their low molecular variation, which suggests rapid diversification immediately following the origin of the group (Bogler and Simpson, 1995, 1996; Eguiarte et al., 2000; Bogler et al., 2006; Good-Avila et al., 2006; Smith et al., 2008; Archibald et al., 2015; Heyduk et al., 2016; McKain et al., 2016; Flores-Abreu et al., 2019). Previous studies have failed to recover *Agave* as a monophyletic genus, since it usually nests within the clade *Agave sensu lato*, along with *Manfreda*, *Polianthes*, and *Prochnyanthes* (Bogler and Simpson, 1995, 1996; Eguiarte et al., 2000; Bogler et al., 2006; Good-Avila et al., 2006; Flores-Abreu et al., 2019).

As mentioned above, the second diversification of *Agave sensu lato* is considered to be the result of the pressures imposed by pollinators (Good-Avila et al., 2006; Rocha et al., 2006; Flores-Abreu et al., 2019). It has been shown that pollinators influence the reproductive and phenological traits of various groups of plants, for instance selecting for synchronization of the flowering time of individuals of a given plant species (Percival and Morgan, 1965; Van der Niet and Johnson, 2012; Lagomarsino et al., 2016). Gentry (1982) divided the agaves based on their type of inflorescence into two subgenera, following Berger (1915), *Agave* subgenus, with paniculate inflorescences and *Littaea* subgenus, with spike inflorescences. Chiropterophily syndrome was attributed to species presenting paniculate inflorescences, whereas species presenting spike inflorescences were considered to be pollinated exclusively by insects (Schaffer and Schaffer, 1977). However, subsequent studies showed that, regardless of the shape of the inflorescence, the pool of *Agave* pollinators can be broad (Arizaga et al., 2000; Silva-Montellano and Eguiarte, 2003; Rocha et al., 2005, 2006; Trejo-Salazar et al., 2015). One of the proposed scenarios to explain the evolutionary history of the *Agave* pollination syndrome is that they evolved from a group of Asparagaceae with moth pollination, which later specialized in a bat pollination syndrome (Smith et al., 2008; McKain et al., 2016). It is likely that, in the specific case of the agaves, they went from being pollinated by insects to become specialized for bat pollination, as bats might have exerted pressure on the agaves and selected for larger inflorescences and individuals producing a greater amount of nectar (Schaffer and Schaffer, 1977, 1979; Eguiarte et al., 2000; Rocha et al., 2005). Coevolution studies report a similar time of origin for both Asparagaceae and the Phyllostomidae (Chiroptera) family, to which the genus *Leptonycteris* belongs, that is the primary pollinator of many *Agave* species (Flores-Abreu et al., 2019).

The aim of the present study is to carry out a phylogenetic reconstruction of the *Agave* genus, by substantially increasing the taxonomic sampling and selecting the appropriate molecular markers, relative to previous studies, in order to obtain a higher resolution level and support values. Our taxonomic sample included 83 species of *Agave sensu lato* (*Agave sensu stricto* clade + *Manfreda* + *Polianthes* and *Prochnyanthes*); from this, 74 species correspond to *Agave sensu stricto*, this sampling includes at least one member of each of the morphological groups proposed by Gentry (1982). Table 1 describes the subgenus and group to which each species analyzed in this study belongs. For this, two types of markers were used: chloroplast and nuclear, the latter had only been used at the intergeneric level or in the limited number of species within *Agave* (Bogler and Simpson, 1996; Lledías et al., 2020). Furthermore, Bayesian approaches were used to estimate the time of divergence of the main groups that conform *Agave sensu lato* and to reconstruct the ancestral character states for the type of inflorescence in order to trace the evolutionary history of this character and to assess its potential importance in the diversification of the group.

**TABLE 1:** List of taxa included in the phylogenetic inference analyses.

**Subgenus *Littaea***		
Littaea group	No. species sampled	Sampled species names
Amolae (8)	4	*attenuata*, *nizandensis*, *ocahui*, *vilmoriniana*
Marginatae (27)	16	*convallis*, *chazaroi*, *doctorensis*, *ghiesbreghtii*, *glomeruliflora*, *horrida*, *kerchovei*, *lechuguilla*, *montium*, *peacockii*, *pelona*, *pintilla*, *titanota*, *triangularis*, *univittata*, *victoria-reginae*
Filiferae (7)	3	*felgeri*, *multifilifera*, *schidigera*
Parviflorae (4)	3	*parviflora*, *polianthiflora*, *schottii*
Polycephalae (5)	2	*chiapensis*, *pendula*
Urceolatae (2)	1	*arizonica*
Striatae (5)	4	*dasylirioides*, *striata*, *rzedowskiana*, *petrophila*
Choripetalae (3)	3	*bracteosa*, *ellemeetiana*, *guiengola*
Total 61	36	59% of total
**Subgenus *Agave***		

**Agave group**	**No. species sampled**	**Sampled species names**

Americanae (6)	4	*americana*, *lurida*, *scabra* (*asperrima*), *scaposa*
Antillanae (14)	1	*antillarum*
Campaniflorae (3)	2	*aurea*, *capensis*
Ditepalae (12)	5	*applanata*, *colorata*, *delamateri*, *phillipsiana*, *wocomahi*
Hiemiflorae (13)	4	*atrovirens*, *isthmensis*, *potatorum*, *seemanniana*
Crenatae (7)	3	*cupreata*, *inaequidens*, *maximiliana*
Deserticolae (10)	4	*cerulata*, *deserti*, *mckelveyana*, *sobria*
Marmoratae (4)	3	*grijalvensis*, *marmorata*, *zebra*
Parryanae (8)	3	*gentryi*, *ovatifolia*, *parryi*
Rigidae (13)	4	*angustifolia*, *datylio*, *rhodacantha*, *tequilana*
Salmianae (5)	1	*salmiana*
Sisalanae (5)	2	*desmettiana*, *sisalana*
Umbeliflorae (2)	1	*shawii*
Total 102	37	36% of total

***Agave sensu lato***	**No. species sampled**	**Sampled species names**

*Manfreda* (37)	4	*scabra*, *virginica*, *umbrophila*, *hauniensis*
*Polianthes* (14)	4	*bicolor*, *densiflora*, *geminiflora*, *longiflora*
*Prochnyanthes* (1)	1	*mexicana*
Total 52	9	22% of total

**Outgroup**	**No. species sampled**	**Sampled species names**

*Dasylirion*	3	*wheeleri*, *texanum*, *longissimum*
*Chlorogalum*	2	*parviflorum*, *purpureum*
*Hesperoyucca*	1	*whipplei*
*Hesperaloe*	3	*changii*, *parviflora*, *nocturna*
*Yucca*	5	*filifera*, *thompsoniana*, *linearifolia*, *madrensis*, *brevifolia*
*Camassia*	2	*quamash*, *leichtlinii*
*Furcraea*	4	*longaeva*, *martinezii*, *guatemalensis*, *pubescens*
*Beschorneria*	4	*calcicola*, *rigida*, *albiflora*, *yuccoides*
Total	24	

*We followed the infrageneric classifications for Agave proposed by Gentry (1982) and Álvarez de Zayas (1995). The numbers between parentheses represent the number of currently known species that conform each one of the subgeneric groups analyzed. Prochnyanthes, Polianthes, and Manfreda species numbers are in agreement with Solano and Feria (2007),Castro-Castro et al. (2010), and Castro-Castro et al. (2018), respectively.*

## Materials and Methods

### Taxon Sampling, DNA Isolation, and Amplification

A total of 107 species of the Asparagaceae family were sampled for the phylogenetic analysis from which 83 correspond to the *Agave sensu lato* clade (74 *Agave sensu stricto* + 4 *Polianthes* + 4 *Manfreda* and 1 *Prochnyanthes*, which together represent 30% of the species that conform the genus) and as outgroups the genera *Dasylirion* (3), *Hesperoyucca* (1), *Chlorogalum* (2), *Camassia* (2), *Hesperaloe* (3), *Yucca* (5), *Beschorneria* (4), and *Furcraea* (4).

The concatenated matrix used for the chloroplast phylogeny included sequences of mat*K* (1,436 bp), *rps*16 (835 bp), *trn*H-*psb*A (561 bp), and *rpl*32-*trn*L (838 bp) from 43 *Agave sensu stricto* species + 2 *Manfreda* + 1 *Polianthes* and *Furcraea* (2) + *Beschorneria* (1) and *Yucca* (1) species as outgroups. For the internal transcribed spacer (ITS) data set, we analyzed 577 bp from 72 *Agave sensu stricto* species + 4 *Manfreda* + 4 *Polianthes* + 1 *Prochnyanthes* and *Dasylirion* (3), *Hesperoyucca* (1), *Chlorogalum* (2), *Camassia* (2), *Hesperaloe* (3), *Yucca* (5), *Beschorneria* (4), and *Furcraea* (4) as outgroups. All newly generated nucleotide sequences for this study were deposited in the NCBI GenBank.

Total genomic DNA was isolated from silica-dried leaf materials and herbarium specimens, using a modification of the CTAB method (Doyle and Doyle, 1987). We used polymerase chain reactions (PCR) to amplify five gene regions, including four plastid DNA regions: mat*K*, *rps*16, *trn*H-*psb*A (Shaw et al., 2005), *rpl*32-*trn*L (Shaw et al., 2007), and the nuclear *ITS*1–*ITS*2 region (Bogler and Simpson, 1996). Amplified products were purified and sequenced by Macrogen, United States, and the complementary chains were visualized and assembled using the DNA Baser version 2.9.97 program (HeracleSoftware). The resulting sequences were aligned with MAFFT (Katoh and Standley, 2013), followed by manual adjustment in PhyDE (Müller et al., 2006). The accession numbers of the sequences obtained in this study and the ones downloaded from the GenBank data base are available as Supplementary Table 1.

### Phylogenetic Reconstructions

The best maximum likelihood (ML) tree for the concatenated matrix from plastid regions was constructed using RAxML (Stamatakis, 2014). We conducted an exhaustive search using PartitionFinder2 (Lanfear et al., 2016) to select the appropriate partitioning scheme for our chloroplast matrix. We provided PartitionFinder2 with subsets for each region, and for the two coding regions, we provided subsets for each nucleotide position. Under the Bayesian Information Criterion (BIC), the “greedy” algorithm, and models = all, PartitionFinder2 identified two partitions: the first partition corresponded to the intron *rps16*, and its best substitution model was the GTR + I model. The second partition included the intergenic spacers *matK* + *trnH-psbA* + *rpl32-trnL*, and the best substitution model was the TrN + I model. The best substitution model for each partition was corroborated in jModelTest (Darriba et al., 2012) under a BIC and then used in the phylogenetic analyses. The analyses were run for 10,000 generations with 1,000 bootstrap replicates, and *Yucca filifera* was specified as outgroup.

For the ITS matrix, the best model was selected based on BIC implemented in jModelTest (Darriba et al., 2012). We ran the ML analyses implementing a GTR + G model for 10,000 generations with 1,000 bootstrap replicates and specifying *Dasylirion* clade as the outgroup.

A Bayesian phylogenetic tree was reconstructed using MrBayes 3.2.2 (Ronquist and Huelsenbeck, 2003). The best substitution model for each partition set was selected using a reversible-jump strategy (Huelsenbeck et al., 2003), and rate heterogeny was modeled with a gamma distribution (Huelsenbeck and Rannala, 2004). Two independent runs with four chains (three heated and one cold) were conducted concurrently for 20,000,000 generations and sampling every 1,000 generations. When the estimated sample size (ESS) value exceeded 200 and the potential scale reduction factor (PSRF) was close to 1.0, it was considered that convergence of the chains occurred. The 25% samples were discarded as burn-in.

### Estimation of Divergence Times and Ancestral State Reconstruction

Bayesian age estimation for the divergence of internal nodes was conducted under an “uncorrelated relaxed clock” model with a lognormal distribution and the tree Birth–Death model in BEAST v2 (Bouckaert et al., 2014). The root node was calibrated under a lognormal distribution, with a mean of 62.49 Ma, which corresponds to the age of the order Asparagales estimated by Magallón et al. (2015). A second point of calibration was the stem age of *Yucca*, with a lognormal distribution and a mean of 14.2 Ma; this includes the age of the strata of the fossil *Protoyucca shadishii* from the middle Miocene, which is considered as being closely related to the *Yucca* genus (Tidwell and Parker, 1990; Wikstrom et al., 2001), and corresponds with previous molecular estimates for the divergence of the *Yucca* clade (Good-Avila et al., 2006; McKain et al., 2016; Flores-Abreu et al., 2019). The analysis was run for 200,000,000 generations sampling every 20,000 from which 25% was discarded as burn-in. The molecular clock analyses were conducted in the CIPRES Science Gateway (Miller et al., 2010). Log outputs of the BEAST analyses were evaluated with tracer v1.5 (Rambaut et al., 2018). Files containing the sampled trees of each MCMC run were combined using LogCombiner v1.7.5, annotated using TreeAnnotator v1.7.5 (Helfrich et al., 2018), and visualized using FigTree v1.4.0.

Inference of the ancestral states for the inflorescence type in the species included in the analysis was based on descriptions and morphological studies (Gentry, 1982; Carrillo-Reyes et al., 2003). The discrete trait inflorescence was coded as a binary character, in which spike inflorescence = 0 and panicle inflorescence = 1. Reconstruction was based on our Bayesian posterior random sample of 500 post burn-in topologies obtained with BEAST v2.0. The ancestral inflorescence type of key nodes from the *Agave sensu lato* clade was reconstructed using the BayesMultistate model as implemented in BayesTraits 3.0.2 (Pagel et al., 2004). Initially, a ML analysis was run to derive empirical priors. After setting these priors (uniform distribution 0–10), a Bayesian inference (BI) analysis was performed using a reversible-jump Markov Chain Monte Carlo (rjMCMC) for 5 million generations, sampling every 10,000 generations and discarding the first 25% as burn-in. The convergence of the chains was verified in trace plots and ESS values. The results of BayesTraits were processed using the same script as in Harrington and Reeder (2017), in which we can graph the probability of each character state for that node and the probability of no node existence.

### Diversification Rate Analyses

To analyze the diversification among agaves, we used a Bayesian analysis of macroevolutionary mixtures (BAMM) v2.5.0 software (Rabosky et al., 2014) for (R Studio Team, 2020). Priors were obtained with BAMMtools by providing the BEAST maximum clade credibility tree and total species number across the *Agave sensu lato* clade. It is well-known that incomplete taxon sampling can bias analyses of speciation and extinction from phylogenetic trees. BAMM accounts for incomplete sampling by analyzing the proportion of tips sampled for a given clade under the assumption that species are missing at random from the tree; species number was obtained from published sources.

Diversification rates were inferred using the function “speciation–extinction” of BAMM, which allows detecting rate shifts (assumed a compound Poisson process in the phylogeny) along tree branches. The evolutionary rate parameters used were: expected number of shift = 1.0, lamdaIntPrior = 1.0, lambdaShiftPrior = 0.05, and muInitPrior = 1.0. BAMM uses rjMCMC to explore the distinct evolutionary models that best explain the whole diversification of the clade. The analysis was conducted by concurrently running two independent chains for 20,000,000 generations and assuming convergence of the chains when the ESS value exceeded 200. For diversification analyses, we retrieved the configuration of rate shifts with the highest posterior probability through the “getBestShiftConfiguration” function of BAMMtools. These configurations were depicted as phylorate plots, which represent the analyzed phylogeny with its branches colored to reflect the instantaneous diversification rate. Rates-through-time plots were generated for speciation (λ), extinction (μ), and diversification (*r*) for both *Agave sensu lato* clade and other groups identified as having significant rate shifts in speciation. We used the functions getCladeRates to obtain estimates of the speciation rate (λ) and an extinction rate (μ) for a specific clade.

## Results

### Phylogenetic Analysis

For the chloroplast phylogeny, a total of 3,670 bp from 46 *Agave sensu lato* species were analyzed. The data set contained a total of 29 variable sites, from which 19 were informative. The BI and ML reconstruction were congruent and recovered some of the morphological delimited genera. The *Furcraea* and *Beschorneria* species included in the analyses were grouped together. The *Agave sensu lato* group included *Manfreda*, *Polianthes*, and *Agave sensu stricto* and appeared as monophyletic (0.87 PP/95.3% BS), whereas *Agave sensu stricto* was not monophyletic. ML and BI trees are shown in Supplementary Figures S1, S2.

**FIGURE 1:**
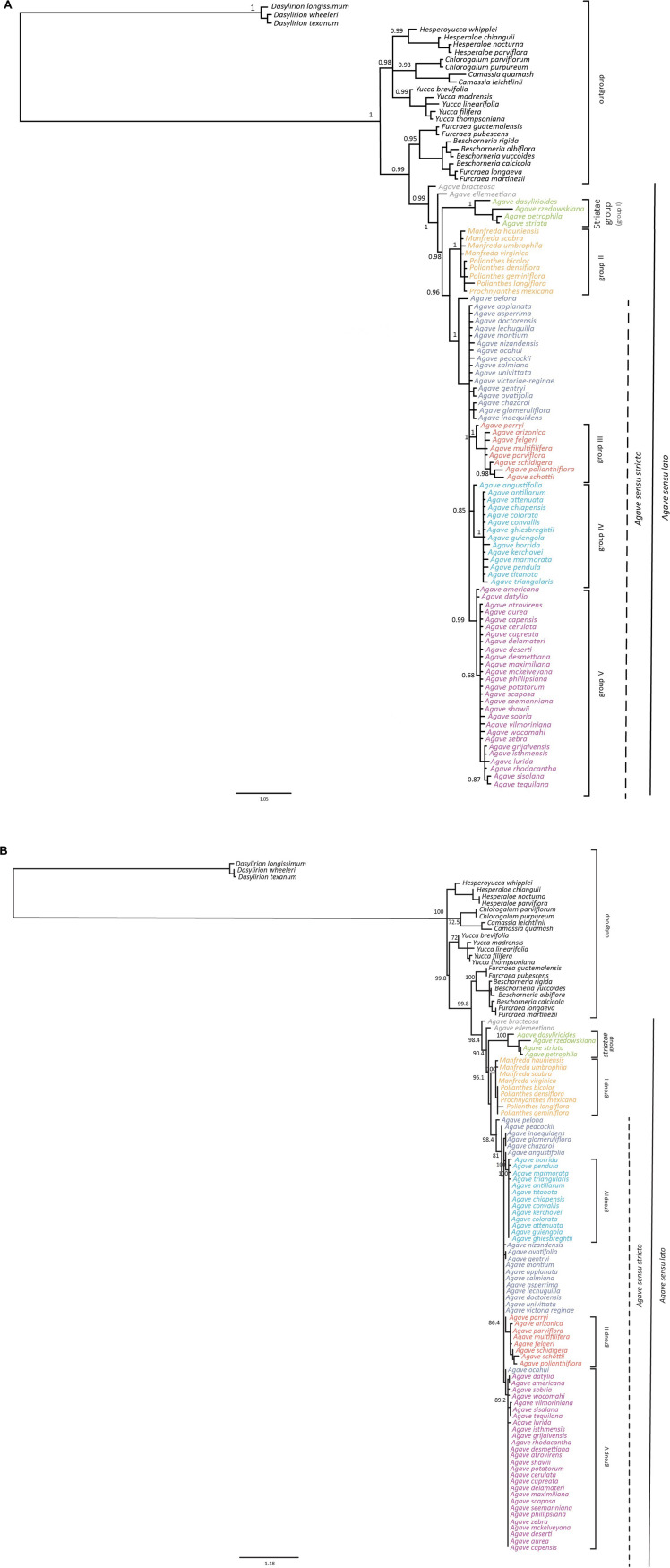
**(A)** Molecular phylogeny of *Agave sensu lato* using *Dasylirion* as an outgroup reconstructed from the ITS sequences and using a Bayesian inference analysis. The numbers next to the nodes indicate the posterior probability values above 0.70. **(B)** Molecular phylogeny of *Agave sensu lato* using *Dasylirion* as an outgroup reconstructed from the ITS sequences and using a maximum likelihood analysis. Numbers next to the nodes are bootstrap values generated from running 1,000 replicates of the trees; only values above 70% are shown.

For the ITS data set, we analyzed 577 bp for the total 105 species described in “Materials and Methods” section, and the matrix contained a total of 168 variable sites of which 155 were informative. Both analyses (ML and BI) resulted in congruent topologies (Figures 1A,B). BI best resolved the earliest-diverging clades: one composed by the *Hesperaloe*/*Hesperoyucca* + *Chlorogalum*/*Camassia* + *Yucca* groups and a second one formed by the *Furcraea*/*Beschorneria* + *Agave sensu lato* groups. However, the *Hesperaloe*/*Hesperoyucca*, *Chlorogalum*/*Camassia*, and *Yucca* groups were not resolved. The ML tree was also unresolved for the *Hesperaloe*/*Hesperoyucca*, *Chlorogalum*/*Camassia*, and *Yucca* + *Furcraea*/*Beschorneria* + *Agave sensu lato* clades. Nonetheless, in both analyses, BI and ML trees were in agreement that the *Yucca* group is independent to the lineage leading to the *Furcraea*/*Beschorneria* + *Agave sensu lato* groups. The *Furcraea*–*Beschorneria* clade (0.95 PP/100% BS) came out as a sister group to *Agave sensu lato* in both analyses (0.99 PP/99.8% BS). However, the *Agave sensu stricto* group is paraphyletic with respect to *Manfreda*, *Prochnyanthes* plus *Polianthes*, that together constitute a clade with high support value (1 PP/100% BS) (Figures 1A,B).

Two species of the *Agave sensu lato* clade, *Agave ellemeetiana* (1 PP/98.4 BS) and *Agave bracteosa* (0.99 PP/98.4 BS) belonging to the Choripetalae group, consistently emerged early in the evolution of the group using either BI or ML methods forming a paraphyletic grade (Figures 1A,B).

The clade that conformed by *Agave dasylirioides*, *Agave striata*, *Agave rzedowskiana*, and *Agave petrophila* was well supported by our analyses (1.0 PP/100% BS) and was clearly separated from the clade that we will name here as *Agave sensu stricto* and from Group II containing *Manfreda*, *Polianthes*, and *Prochnyanthes* (1.0 PP/100 BS%) (Figures 1A,B).

Inside the *Agave sensu stricto* clade (1.0 PP/98.4% BS), *Agave pelona* (1.0 PP/98.4% BS) was positioned as the sister of the *Agave sensu stricto* clade (Figures 1A,B). *Agave sensu stricto* was conformed in our analyses by three distinctive groups: III, IV, and V (Figures 1A,B).

Group III (1 PP/86.4% BS) comprises *Agave parryi*, *Agave arizonica*, *Agave felgeri*, *Agave multifilifera*, *Agave parviflora*, *Agave schidigera*, *Agave polianthiflora*, and *Agave schottii*.

Group IV (0.85 PP/100% BS) included *Agave angustifolia* and a subgroup formed by *Agave antillarum*, *Agave attenuata*, *Agave chiapensis*, *Agave colorata*, *Agave convallis*, *Agave ghiesbreghtii*, *Agave guiengola*, *Agave horrida*, *Agave kerchovei*, *Agave marmorata*, *Agave pendula*, *Agave titanota*, and *Agave triangularis*.

Group V (0.99 PP/89.2% BS) was composed of *Agave americana*, *Agave datylio*, *Agave atrovirens*, *Agave aurea*, *Agave capensis*, *Agave cerulata*, *Agave cupreata*, *Agave delamateri*, *Agave deserti*, *Agave desmettiana*, *Agave maximiliana*, *Agave mckelveyana*, *Agave phillipsiana*, *Agave potatorum*, *Agave scaposa*, *Agave seemanniana*, *Agave shawii*, *Agave sobria*, *Agave vilmoriniana*, *Agave wocomahi*, *Agave zebra*, *Agave grijalvensis*, *Agave isthmensis*, *Agave lurida*, *Agave rhodacantha*, *Agave sisalana*, and *Agave tequilana* (Figures 1A,B).

Other agave species that also belong to *Agave sensu stricto* were not part of the above-mentioned strongly supported clades and were found paraphyletic to these clades, both by BI and ML (Figures 1A,B), including *Agave applanata*, *Agave asperrima*, *Agave doctorensis*, *Agave lechuguilla*, *Agave montium*, *Agave nizandensis*, *Agave ocahui*, *Agave peacockii*, *Agave salmiana*, *Agave univittata*, *Agave victoria-reginae*, *Agave gentryi*, *Agave ovatifolia*, *Agave chazaroi*, *Agave glomeruliflora*, and *Agave inaequidens*. The only discrepancy was *A. ocahui* that according to ML is a Group V member, but not in BI.

### Divergence Times

**FIGURE 2:**
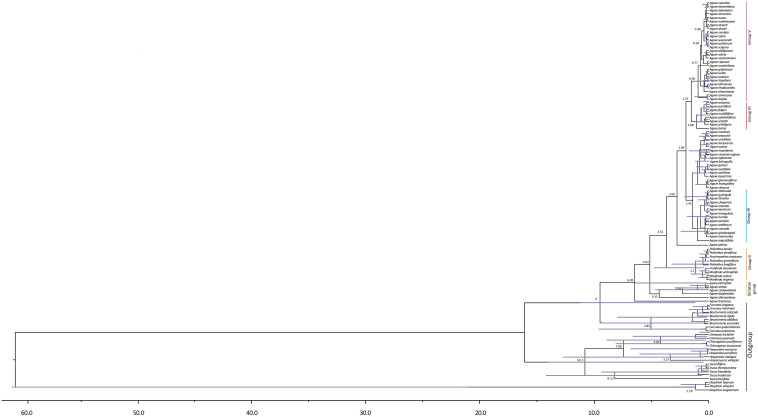
Timing of *Agave sensu lato* diversification. Chronogram derived from the maximum clade credibility tree estimated with BEAST. Numbers indicate mean divergence times and mean ages, and the 95% highest posterior densities (HPD) are represented with bars.

Our analyses based on ITS sequences estimated the divergence of the *Yucca* group from the *Hesperaloe*/*Hesperoyucca* and *Camassia*/*Chlorogalum* groups with a stem age at 14.2, but a far more recent crown age for *Yucca* at 8.52 Ma; this should be considered with caution, given that only five species of this genus were analyzed. The paraphyletic group that includes *Hesperaloe*/*Hesperoyucca* and *Camassia*/*Chlorogalum* had a more recent stem age of 7.09 Ma. Finally, the *Beschorneria*/*Furcraea* clade presented a stem age of 9 Ma and a crown age of 4.8 Ma (Figure 2).

For *Agave sensu lato*, the stem age was 9 Ma and a crown age of 6.18 Ma. Noticeably, within the *Agave sensu lato* clade, the Striatae group diverged earlier than the other clades, with a stem age of 4.15 Ma and a crown age of 2.24 Ma (Figure 2). The paraphyletic group including *Manfreda*, *Polianthes*, and *Prochnyanthes* had a stem age of 3.55 Ma and a crown age of 1.2 Ma. For the *Agave sensu stricto* clade, we estimated a stem age of 3.55 Ma and a crown age of 2.68 Ma. For Group III, we obtained a stem age of 1.53 Ma and a crown age of 1.08 Ma; for Group IV, a stem age of 1.41 Ma and a crown age of 1.04 Ma; and finally, for Group V, a stem age of 1.53 Ma and a crown age of 0.96 Ma (Table 2).

**TABLE 2:** Significant shifts in diversification rate where *r* is the diversification rate = (λ − μ).

Clade	Stem age (Ma)	Speciation rate (λ) (species/Mya)	Extinction rate (μ) (species/Mya)	Diversification rate (*r*) (species/Mya)	Event shift rate
*Yucca*	14.2	1.64	1.52	0.12	
*Furcraea–Beschorneria*	9	1.65	1.54	0.11	
Striatae	4.15	1.76	1.58	0.18	
*Manfreda–Polianthes–Prochnyanthes*	3.55	3.22	2.39	0.82	
*Agave sensu lato*	9	3.66	2.16	1.50	
*Agave sensu stricto*	3.55	5.67	2.69	2.98	
Group III	1.53	6.05	2.74	3.31	
Group IV	1.41	6.15	2.74	3.41	
Group V	1.53	6.15	2.73	3.41	
Shift 1: *Agave bracteosa* + *Agave sensu lato*	6.18	Start 1.65 End 4.10	Start 1.54 End 2.30	Start 0.11 End 1.8	Increase speciation rate
Shift 2: *Agave sensu stricto*	2.68	Start 3.22 End 6.04	Start 2.39 End 2.73	Start 0.82 End 3.31	Increase speciation rate

*Shift 1 and Shift 2 indicate the reconstructed start rate versus end rate.*

### Inflorescence Reconstruction Analysis

**FIGURE 3:**
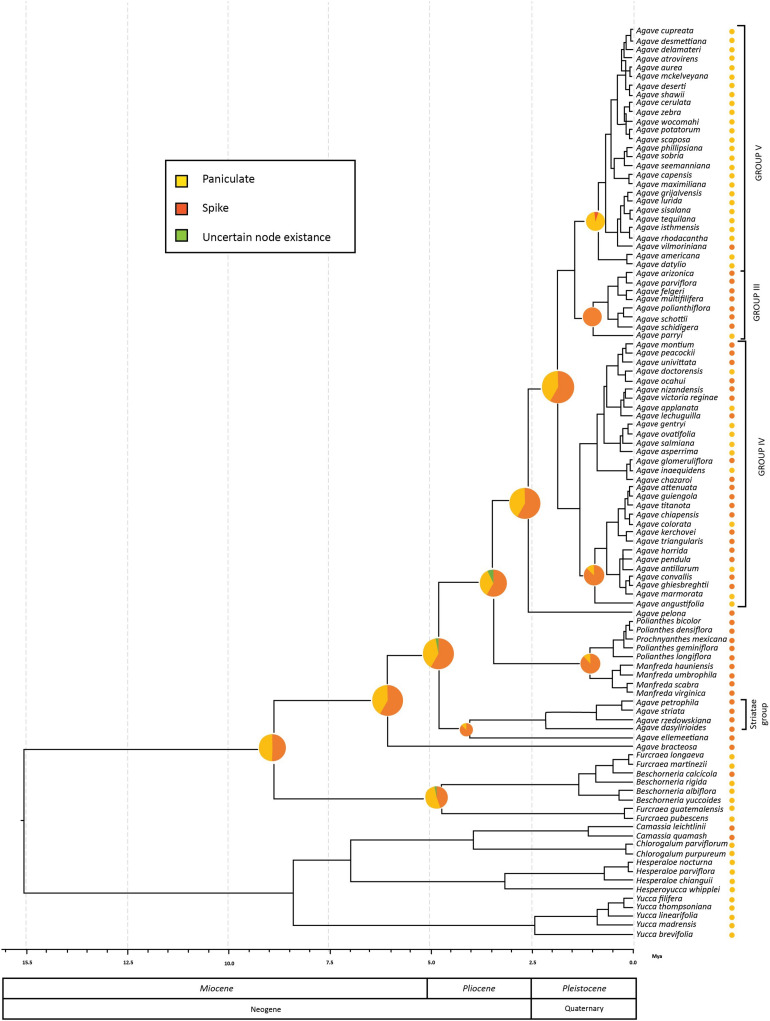
Ancestral reconstruction of inflorescence type mapped onto *Agave sensu lato* chronogram resulting from BEAST analyses. Circles next to the tips taxa are coded to represent the actual inflorescence type. The pie charts show the probability values generated from BayesTraits for the ancestral inflorescence type reconstructed at each node.

The reconstruction of the inflorescence types showed ambiguous results in the sense that it was not clear if the common ancestor of the *Furcraea*–*Beschorneria* and *Agave sensu lato* clades had a paniculated inflorescence or not. In contrast, for the common ancestor of the *Agave sensu stricto* clade, we found a higher probability (75% of the reconstructions) for the presence of a spike inflorescence (Figure 3).

The reconstructions for the common ancestor of two species with early divergence from the *Agave sensu lato* clade (*A. bracteosa* and *A. ellemeetiana*), as well as Striatae clade (Figure 3), indicated that they may have presented a spike inflorescence (reconstruction probabilities of 80 and 60%, respectively, for each group), which was also the case for the ancestor of the paraphyletic group containing *Manfreda*, *Polianthes*, and *Prochnyanthes* (93% of the reconstructions).

Within the *Agave sensu stricto* clade, *A. pelona* is the sister taxa to the rest of the group and presents a spike inflorescence, in agreement with 75% of the reconstructions for the common ancestor of the *Agave sensu stricto* clade having spike inflorescences. The common ancestor for Group III most likely showed a spike inflorescence (98%). In contrast, for Group V, the one with the most recent origin, the common ancestor, and the extant species presented a panicle inflorescence (98%), with the exception of *A. vilmoriniana*, which reverted to a spike inflorescence (Figure 3).

### Diversification Rates

Our estimate of diversification rate (*r*) using the ITS data estimated for *Agave sensu lato* was 1.50 species/Myr, with a speciation rate of λ = 3.66 species/Myr and an extinction rate of μ = 2.16 species/Myr (more information in Table 2). In contrast, the diversification rate in the *Yucca* clade was an order of magnitude lower, *r* = 0.12 species/Myr, with λ = 1.64 species/Myr and μ = 1.52 species/Myr, similar to the estimated rates for the *Furcraea*–*Beschorneria* clade *r* = 0.11 species/Myr, λ = 1.65 species/Myr, μ = 1.54 species/Myr (Table 2).

**FIGURE 4:**
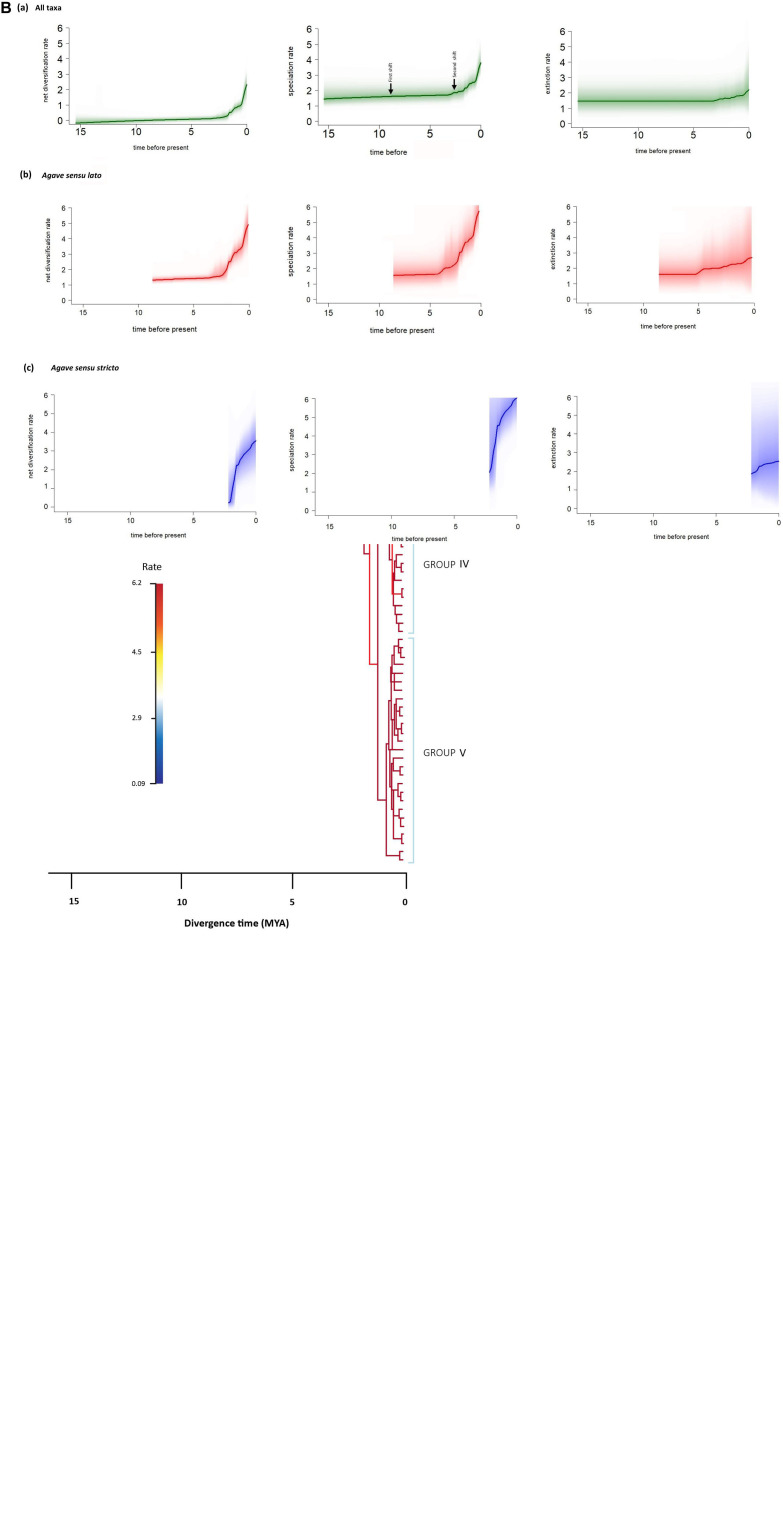
BAMM analysis of rate shift configurations and diversification within *Agave sensu lato*. **(A)** Rate shift configurations with the two highest posterior probabilities from the 95% credible set are indicated with red circles, and branches are colored according to median net diversification rates (cool colors = slow, warm = fast). The first shift corresponds to the stem of *A. bracteosa* branch and the sister group *Agave sensu lato* clade, and the second shift corresponds to the *Agave sensu stricto* clade. Group III corresponds to the group including *A. parryi*, Group IV corresponds to the group including *A. angustifolia*, and Group V corresponds to the group including *A. americana* and *A. datylio*. **(B)** Evolutionary rates through time plots for (a) all taxa sampled, (b) *Agave sensu lato*, and (c) *Agave sensu stricto*; solid lines denote the mean of each rate-through-time curve across all agaves, and the shading intensity of the colored line for each species reflects the relative probability of a given diversification trajectory, with upper and lower bounds representing the 90% Bayesian credible interval on the distribution of rates through time.

BAMM identifies configurations of the rate shifts, that is, the sets of shifts that are identified together and enables to compute the relative probability of those configurations. The rate shift configuration analyses exhibited two main changes (Figure 4A). The first shift detected an increase in speciation rate, *r* = 1.80 species/Myr, λ = 4.10 species/Myr; this branch corresponds to the stem age of *A. bracteosa* and its sister group the *Agave sensu lato* clade at 6.18 Mya. The second shift also detected an increase in speciation rate (*r* = 3.31 species/My, λ = 6.04 species/Myr); this shift is located at the branch of the *Agave sensu stricto* clade at 4.91 Mya (see Table 2). We obtained the rate-through-time plots of speciation, extinction, and net diversification rates for all taxa included, as well as for *Agave sensu lato* and *Agave sensu stricto* clades, with BAMM in order to examine rate variation through time (Figure 4B).

## Discussion

The number of variable sites found for the plastid data set was low (29 variable from a total of 3,670 sites), which is congruent with previous analyses that included plastid markers for the *Agave sensu lato* clade (Flores-Abreu et al., 2019). The phylograms obtained previously in different studies detected clades in which *Manfreda*, *Polianthes*, and *Prochnyanthes* species are placed within the *Agave sensu stricto* group (Good-Avila et al., 2006; Flores-Abreu et al., 2019). Indeed, *Agave sensu lato* is a group that has been difficult to taxonomically classify because of overlapping variation of morphological characters between species (Gentry, 1982; García-Mendoza, 2002), which could be due to the recent origin of the group, the recent diversification events, as well as permissive hybridization between species and a long generation time (McKain et al., 2016).

The nuclear data set has more variable sites (169 from a total of 577 bp) than the plastid data set and showed higher resolution and support values. The first phylogenetic study from ITS markers for the clade by Bogler and Simpson (1996) only included nine species, whereas Eguiarte et al. (2000) included 10 species for the *Agave sensu lato* clade. In the aligned *ITS2* sequence, there is a large deletion shared by *Yucca* species, first noticed by Bogler and Simpson (1996). The groups within *Agave sensu lato* detected in our study are consistent with these previous studies (Bogler and Simpson, 1996; Eguiarte et al., 2000, 2006). In our analysis (BI and ML), the species *A. bracteosa* and *A. ellemeetiana* of the Choripetalae group can be observed, as well as the Striatae group as paraphyletic with respect to the clade *Agave sensu stricto*. It is possible that the marker used in our study, the nuclear marker ITS, allowed us to trace a different history to the one obtained using chloroplast markers. Moreover, it is known that a greater taxonomic sampling can influence the results of phylogenetic analyses.

The phylogenetic results of certain groups, such as the herbaceous, polycarpic (iteroparous) clade *Manfreda*–*Polianthes*–*Prochnyanthes*, are interesting, given that the *Polianth*es and *Prochnyanthes* are paraphyletic with respect to *Manfreda*. This nesting was also reported in previous studies (see Bogler and Simpson, 1996; Eguiarte et al., 2000; Bogler et al., 2006). The Striatae group proposed by Gentry (1982) originally consisted of only three species: *A. striata*, *A. dasylirioides* (both included in this study), and *A. stricta* (closely related to *A. striata*, see Scheinvar et al., 2017 and Figure 2 therein). Subsequently, other species that belong to this group have been described: *A. petrophila* (García-Mendoza and Salas, 1998), *A. gracielae* (Galván and Zamudio, 2013), *A. cremnophila* (Starr et al., 2018), *A. lexii* (García-Morales and García-Jiménez, 2019), *A. rzedowskiana* (Carrillo-Reyes et al., 2003), *A. tenuifolia* (Galván and Zamudio, 2013), *A. albopilosa* (Cabral Cordero et al., 2007), and *Agave kavandivi* (García-Mendoza and Chávez-Rendón, 2013), which conform a total of 11 species, are all endemic to Mexico. *A. petrophila* and *A. rzedowskiana* were analyzed in our study. One of the most important characters of the Striatae group is the presence of finely denticulated leaf margins. It is also relevant to mention that this group seems to be formed by polycarpic (iteroparous) species, in contrast with most *A. sensu stricto* species, that are usually monocarpic (semelparous). The Striatae group seems to be less frequently pollinated by bats than the other species in the *A. sensu stricto* group (Rocha et al., 2005, 2006). It will be important to include all the species of the Striatae group in the future and corroborate the coherence of this clade.

The two species at the base of the *Agave sensu lato* clade are *A. ellemeetiana* and *A. bracteosa*, which Gentry (1982) defined as part of his Choripetalae group. Gentry (1982) already recognized the uniqueness of this group, stating that their unarmed leaves and discoid floral receptacle are the principal characters that separate these species into another group, noticing that (page 89): “This distinctive flower structure together with the unarmed leaves without terminal spine, could justify removal from *Agave* to a separate genus,” but future formal trait analyses are needed. Gentry (1982) also included *A. guiengola*, because of its virtually tubeless flower and the insertion of the filaments at the base of the flowers, a species that in our analysis is positioned in Group IV of *Agave sensu lato*. This last placement is not surprising, as Gentry (1982, p. 97) himself noted that “Its broad, white, ovate leaves, with their conspicuous coarse teeth, and its monocarpic rather than polycarpic habit, set off *Agave guiengola* from either of the species mentioned (i.e., *A. ellemeetiana* and *A. bracteosa*).” In particular, *A. ellemeetiana* and *A. bracteosa* are interesting species, since they have margins without any type of teeth, whereas the Striatae group presents serrulate margins; the presence of margins without teeth or serrulate is a character present in many of the early divergent species in the phylogeny of the *Agave sensu lato*. For instance, *A. pelona*, which is paraphyletic with respect to the *Agave sensu stricto* clade, was named this way by Gentry (1982) because of the absence of marginal teeth. It would be relevant to make an analysis including the total species within the Striatae group proposed by Gentry (1982), in order to observe if there is a modification in the phylogenetic relationships within this current clade.

*Agave sensu lato* started diversifying at 6.18 Ma according to our crown age estimate. This is congruent with the first significant diversification shift obtained from BAMM, which is at the base of the *Agave sensu lato* clade. This original shift in the diversification rate was previously reported by Good-Avila et al. (2006) and Flores-Abreu et al. (2019). At this point, the mean speciation rate increased (λ = 4.10 species/Myr). This shift rate occurred in the late Miocene period, which is characterized by the emergence of arid biomes in America, resulting in the rise of new mountains, such as the Trans-Mexican Neovolcanic Belt and the Sierra Madre Occidental (Mastretta-Yanes et al., 2015). This emergence caused changes in humidity and wind currents, originating new habitats, which generated new ecological opportunities for several lineages that inhabit these arid areas today (Morán-Zenteno and Wilson, 1994; Good-Avila et al., 2006; Arakaki et al., 2011; De-Nova et al., 2012; Hernández-Hernández et al., 2014). The first rate shift is linked with the origin of the *Agave sensu lato* clade, and it could be the starting point for the diversification of agaves. In *Agave sensu lato*, the earlier divergent groups [i.e., *A. ellemeetiana* and *A. bracteosa* (Choripetalae group) and clades Striatae and *Manfreda*–*Polianthes*–*Prochnyanthes*] have predominant ancestors with spike inflorescences according to our ancestral character state reconstruction, which are commonly pollinated by bees and hawk moths (Eguiarte, 1995; Rocha et al., 2005, 2006). This trend was maintained in the *Agave sensu stricto* clade, where the earlier divergent groups still have spike inflorescences. For instance, Groups III and IV, two of the most recent groups, at 1.2 Ma usually display spike inflorescences, although several conversions toward paniculate inflorescences also occur. This time could have represented a period of transition, when the agaves went from having mainly spike inflorescences to evolving paniculate inflorescences, until reaching the origin of the recent Group V (0.96 Ma), where the predominant character is a paniculate inflorescence.

Are these reconstructed chains of events consistent with what we know about inflorescence development and evolution? Inflorescence architecture is the consequence of developmental programs that dictate inflorescence meristem activity and determine organ topology, geometry, and phenology by means of the regulatory processes affecting meristem identity, size, and maintenance, as well as axillary meristem initiation and organogenesis (Zhang and Zheng, 2014). There is ample evidence that these programs are hormonally and genetically controlled, and the rich diversity in inflorescence architecture in angiosperms is evidence of its enormous plasticity (Harder and Prusinkiewicz, 2013; Zhang and Zheng, 2014). Inflorescence architecture can influence pollination and seed yield, playing important roles in natural selection. Complex, simple, or small architectures solve the problem of attracting specific kinds of pollinators or promote self-pollination (Harder and Prusinkiewicz, 2013). Agavoideae displays varying inflorescence architectures, panicles being more common in *Agave*, *Manfreda*, *Beschorneria*, *Furcraea*, *Hesperaloe*, and *Yucca*, whereas spike or raceme inflorescences are found in *Hesperaloe*, *Polianthes*, *Prochnyanthes*, and also in *Agave* (Aker, 1982; Gentry, 1982; Starr, 1997; García-Mendoza, 2000; Castro-Castro et al., 2010, 2018; Solano et al., 2013; Cházaro-Basáñez and Vázquez-Ramírez, 2015). High plasticity of inflorescence architecture has been more clearly demonstrated in grasses, where molecular switches can significantly increase secondary and tertiary branching, thus changing inflorescence morphology (Zhang and Zheng, 2014). Therefore, inflorescence architecture in Agavoideae can be reasonably considered as homoplastic, given its plastic nature. The underlying natural forces that kept quite stable spike inflorescences in the early-diverging *Agave* groups (Choripetalae and Groups I and II), panicle inflorescences in the late-diverging groups (Group V), and frequent reversions between the two forms (Groups III and IV) remain to be studied.

During the Pliocene and Pleistocene, the agaves had the greatest amount of diversification events. This corresponds to the second rate shift within the stem of *A. pelona* and its sister group *Agave sensu stricto* at 2.68 Ma when we observed an increase in the speciation rate (λ = 6.04 species/Myr). This is concordant with Scheinvar et al. (2017),Scheinvar (2018), and Aguirre-Planter et al. (2020) that suggested that current *Agave sensu lato* distribution and species richness could be related to glaciation and interglacial events during the Pleistocene that caused the expansion and contraction of the species distribution, thus influencing the evolution of agave populations. Three localities of interest during this period are the southern portion of Sierra Sur de Chihuahua, which served as refuge during the last interglacial period, the Sierra Madre del Sur, which is considered as a refuge during the Last Glacial Maximum (21,000–17,000 years), and the California Sierra during both periods. This second increase in the diversification rate is in accordance with a second diversification proposed by Good-Avila et al. (2006), but that was not detected by Flores-Abreu et al. (2019), even though the latter study had a large sample. Good-Avila et al. (2006) suggested that this second shift was related to changes in the pollinators (to bat pollination), but the analyses of Flores-Abreu et al. (2019) falsified this idea, as bat pollination in *Agave* seems to be older. The second shift could represent a secondary adaptation to different climates that permitted the lineages to diversify, including adaptation to more mesic conditions in Central and West Mexico, giving rise, for instance, to the radiation of the *Manfreda*–*Polianthes*–*Prochnyanthes* herbaceous group and of some agave groups that live in less arid environments in central Mexico.

It is clear that agaves keep a close relationship with their pollinators, as exemplified by the large number of agave species that are distributed in the so called “nectar corridors” found in the migratory routes of several bat species (Moreno-Valdez et al., 2004; Trejo-Salazar et al., 2016), in the phenologies of pollen and nectar production (Schaffer and Schaffer, 1977; Nassar et al., 2003), and in the relationship between bat visits and agave reproduction rate and genetic variation, as well as in their function as primary pollinators, although the total list of floral visitors and potential pollinators is wide (Howell and Roth, 1981; Eguiarte and Búrquez, 1987; Arizaga et al., 2000; Slauson, 2000, 2001; Molina-Freaner and Eguiarte, 2003). However, it does not appear to be a strict and tight coevolution process, as in other groups of the Asparagaceae (Flores-Abreu et al., 2019), such as in the *Yucca* family, because few bat species, in particular *Leptonycteris yerbabuenae*, visit and pollinate many species of agave, whereas each species of *Yucca* seems to coevolve with a particular *Tegeticula* moth (Pellmyr, 2003).

On the other hand, the divergence time for clades reported in our study is more recent than the values estimated in previous studies. We consider that this is a consequence of sampling more taxa, as the inferred branch lengths become also shorter. Nonetheless, the periods in which we found increases in speciation rate are congruent with previous studies, as commented above (Good-Avila et al., 2006; Flores-Abreu et al., 2019). It is likely that pollinators, especially bats, have influenced the *Agave* diversification processes and have had a relevant role in selecting the type of inflorescence that agaves currently present.

The diversification rate of *Agave sensu lato* (*r* = 1.50 species/Myr) is clearly higher than that of related groups, such as *Yucca* (*r* = 0.12 species/Myr) and *Furcraea*–*Beschorneria* (*r* = 0.11 species/Myr), values similar to those reported by Flores-Abreu et al. (2019). The mean speciation rate showed that speciation was low during the stem divergence of the group and increased at the base of the *Agave sensu lato* clade. *Agave sensu lato* has a considerably higher diversification rate than average estimates reported for other flowering plants, which range from 0.078 to 0.09 species/Mya (Magallón and Castillo, 2009). The speciation rate seems to increase rapidly at the *Agave sensu stricto* branch (λ = 5.67 species/Myr), continuing to increase in the three main groups that conform the *Agave sensu stricto* clade: Group III with a λ = 6.05 species/Myr, Group IV with a λ = 6.15 species/Myr, and Group V with a λ = 6.15 species/Myr. On the basis of the elevated speciation rate values estimated for the *Agave sensu lato* clades, we can conclude that as a group of recent origin, it is experiencing an intense process of diversification (Table 3).

**TABLE 3:** Age estimation in million years of major divergence events and compared with those found in other studies.

Clade	Stem group mean age	Crown group mean age	Good-Avila et al. (2006)	McKain et al. (2016)	Flores-Abreu et al. (2019)
Asparagaceae			34.2–31.7	41.27	
*Yucca*	14.2	8.52	19.5–11.1	20.10	7.37–16.48
*Furcraea–Beschorneria*	9	4.8		11.41	4.62–12.34
Striatae	4.15	2.24			
*Manfreda–Polianthes–Prochnyanthes*	3.55	1.2			
*Agave sensu lato*	9	6.18	13.1–5.9	3.09	4.62–13.34
*Agave sensu stricto*	4.91	3.55			
Clade III	1.53	1.08			
Clade IV	1.41	1.04			
Clade V	1.53	0.96			

## Data Availability Statement

All sequences generated for this study were deposited in the NCBI GenBank under accession numbers shown in Supplementary Table S1.

## Author Contributions

OJ-B contributed to the laboratory work, data analysis, and drafting of the manuscript. RG-S contributed to the phylogenetic analysis, reconstruction of ancestral state analyses, and design of some figures. SM helped in drafting and correcting sections of the manuscript. AG-M helped in drafting and correcting sections of the manuscript and with the species collection for the analyses. JN-S contributed with ideas for the design of the project, DNA material, and correcting sections of the manuscript. EA-P helped in drafting and correcting sections of the manuscript. LE, as the project leader, designed and coordinated the project and logistics and drafted and corrected the manuscript. All authors contributed to the article and approved the submitted version.

## Conflict of Interest

The authors declare that the research was conducted in the absence of any commercial or financial relationships that could be construed as a potential conflict of interest.

## Supplementary Material

Supplementary Figure 1 Molecular phylogeny of *Agave sensu lato* using *Yucca* as an outgroup reconstructed from the chloroplast data set sequences and using a maximum likelihood analysis. The numbers next to the nodes indicate the posterior probability values above 70%.

Supplementary Figure 2 Molecular phylogeny of *Agave sensu lato* using *Yucca* as an outgroup reconstructed from the chloroplast data set sequences and using a Bayesian inference analysis. The numbers next to the nodes indicate the posterior probability values above 0.70.
