# Supplementary figures and images for: Phylogeny, Diversification Rate, and Divergence Time of Agave sensu lato (Asparagaceae), a Group of Recent Origin in the Process of Diversification
Source: Front Plant Sci. 2020 Nov 9;11:536135. doi: 10.3389/fpls.2020.536135 (PMC7680843; doi:10.3389/fpls.2020.536135)

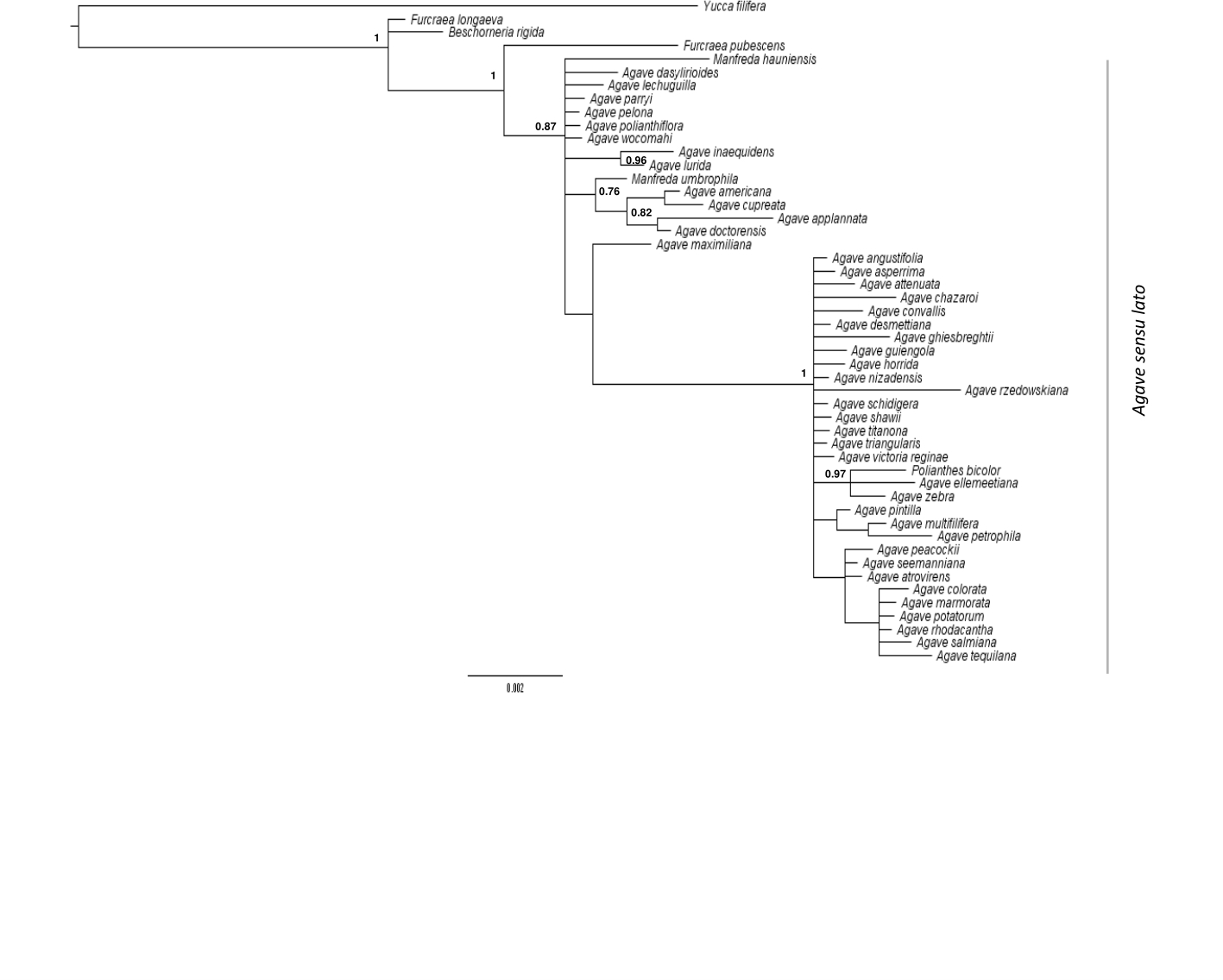

Supplement: Supplementary Figure 1 — Molecular phylogeny of Agave sensu lato using Yucca as an outgroup reconstructed from the chloroplast data set sequences and using a maximum likelihood analysis. The numbers next to the nodes indicate the posterior probability values above 70%. [file Image_1.JPEG]

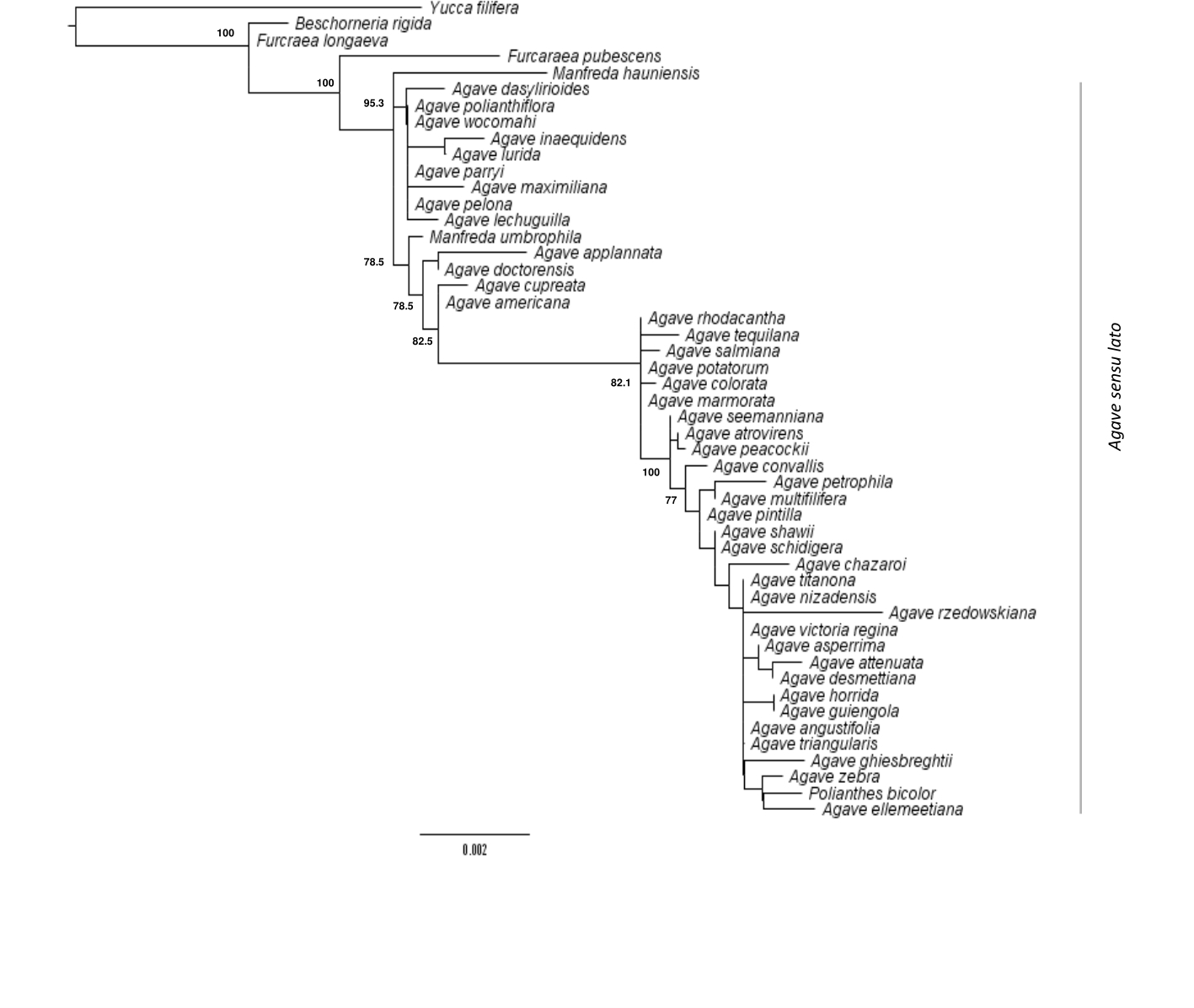

Supplement: Supplementary Figure 2 — Molecular phylogeny of Agave sensu lato using Yucca as an outgroup reconstructed from the chloroplast data set sequences and using a Bayesian inference analysis. The numbers next to the nodes indicate the posterior probability values above 0.70. [file Image_2.JPEG]
