# Supplementary material for: Phylogeny, Diversification Rate, and Divergence Time of Agave sensu lato (Asparagaceae), a Group of Recent Origin in the Process of Diversification
Source: Front Plant Sci. 2020 Nov 9;11:536135. doi: 10.3389/fpls.2020.536135 (PMC7680843; doi:10.3389/fpls.2020.536135)
Supplement: Supplementary file 3 [file Table_1.DOCX]

**Supplementary**

**Table 1. Accessions numbers of GenBank for the sequences implemented in this study**

| Specie | ITS | rpl32-trnl | matK | rps16 | trnH-psbA |
| --- | --- | --- | --- | --- | --- |
| *Dasylirion wheeleri* | U24014.1/U24035.1 |  |  |  |  |
| *Dasylirion texanum* | U24034.1/U24013.1 |  |  |  |  |
| *Dasylirion longissimum* | KC798469.1 |  |  |  |  |
| *Hesperoyucca whipplei* | MF963981.1 |  |  |  |  |
| *Chlorogalum parviflorum* | MF964117.1 |  |  |  |  |
| *Chlorogalum purpureum* | MF963989.1 |  |  |  |  |
| *Camassia quamash* | KP008320.1 |  |  |  |  |
| *Camassia leichtlinii* | KP008304.1 |  |  |  |  |
| *Hesperaloe chiangii* | * |  |  |  |  |
| *Hesperaloe nocturna* | * |  |  |  |  |
| *Hesperaloe parviflora* | MT083804 |  |  |  |  |
| *Yucca madrensis* | * |  |  |  |  |
| *Yucca filifera* | MT083805 | MT250090 | MT250133 |  | MT053314 |
| *Yucca linearifolia* | MT083806 |  |  |  |  |
| *Yucca thompsoniana* | MT083807 |  |  |  |  |
| *Yucca brevifolia* | MF964125.1 |  |  |  |  |
| *Beschorneria albiflora* | MT083808 |  |  |  |  |
| *Beschorneria calcicola* | MT083809 |  |  |  |  |
| *Beschorneria rigida* | MT083810 | MT250093 | MT250134 | MT25170 |  |
| *Beschorneria yuccoides* | U24008.1/U24028.1 |  |  |  |  |
| *Furcraea longaeva* | MT083811 | MT250091 |  | * | MT053315 |
| *Furcraea martinezii* | MT083812 |  |  |  |  |
| *Furcraea guatemalensis* | AM884840.1 |  |  |  |  |
| *Furcraea pubescens* | MT083813 | MT250092 |  | MT250169 | MT053316 |
| *Manfreda hauniensis* | MT083814 | MT250094 | MT250125 | MT250171 | MT053317 |
| *Manfreda scabra* | U23983.1/U24042.1 |  |  |  |  |
| *Manfreda umbrophila* | * | * | * | * | * |
| *Manfreda virginica* | U23984.1/U24043.1 |  |  |  |  |
| *Polianthes bicolor* | MT083815 | MT250095 | MT250136 |  | MT053318 |
| *Polianthes densiflora* | MT083816 |  |  |  |  |
| *Polianthes geminiflora* | MT083817 |  |  |  |  |
| *Polianthes longiflora* | MT083818 |  |  |  |  |
| *Prochnyanthes mexicana* | MT083819 |  |  |  |  |
| *Agave americana* | MT083820 | MT250096 | MT250137 |  | MT053319 |
| *Agave angustifolia* | MT083821 | MT250097 | MT250138 | MT250172 | MT053320 |
| *Agave antillarum* | MT083822 |  |  |  |  |
| *Agave applanata* | MT083823 | MT250098 | MT250139 | MT250173 | * |
| *Agave arizonica* | MT083824 |  |  |  |  |
| *Agave aspérrima* | MT083825 | MT250099 | MT250140 |  | MT053321 |
| *Agave atrovirens* | MT083826 | MT250129 | MT250166 |  |  |
| *Agave attenuata* | MT083827 | MT250100 | MT250141 | MT250174 | MT053322 |
| *Agave aurea* | AM884819.1 |  |  |  |  |
| *Agave bracteosa* | MT083828 |  |  |  |  |
| *Agave capensis* | * |  |  |  |  |
| *Agave cerulata* | MT083829 |  |  |  |  |
| *Agave chazaroi* | * | * | * | * | * |
| *Agave chiapensis* | MT083830 |  |  |  |  |
| *Agave colorata* | MT083831 | MT250101 | MT250142 | MT250175 | MT053323 |
| *Agave convallis* | * | MT250102 |  | MT250176 | MT053324 |
| *Agave cupreata* | MT083832 | MT250103 | MT250143 | MT250177 | MT053325 |
| *Agave dasylirioides* | MT083833 | MT250104 | MT250144 |  | MT053326 |
| *Agave datylio* | AM884827.1 |  |  |  |  |
| *Agave delamateri* | MT083834 |  |  |  |  |
| *Agave deserti* | MT083835 |  |  |  |  |
| *Agave desmenttiana* | * | * | * | * | * |
| *Agave doctorensis* | * | * | * | * | * |
| *Agave ellemeetiana* | MT083836 | MT250105 | MT250145 | MT250178 |  |
| *Agave felgeri* | MT083837 |  |  |  |  |
| *Agave Gentry* | * |  |  |  |  |
| *Agave ghiesbreghtii* | MT083838 | MT250106 | MT250146 | MT250179 | MT053327 |
| *Agave glomeruliflora* | MT083839 |  |  |  |  |
| *Agave grijalvensis* | MT083840 |  |  |  |  |
| *Agave guiengola* | MT083841 | MT250107 | MT250147 |  | MT053328 |
| *Agave horrida* | MT083842 | MT250108 | MT250148 | MT250180 |  |
| *Agave inaequidens* | MT083843 | MT250109 | MT250149 | MT250181 | MT053329 |
| *Agave isthmensis* | MT083844 |  |  |  |  |
| *Agave kerchovei* | MT083845 |  |  |  |  |
| *Agave lechuguilla* | MT083846 | MT250110 | MT250150 | MT250182 | MT053330 |
| *Agave lurida* | MT083847 | MT250111 |  | MT250183 | MT053331 |
| *Agave marmorata* | MT083848 | MT250112 |  | MT250184 | MT053332 |
| *Agave maximiliana* | MT083849 | MT250113 | MT250151 | MT250185 |  |
| *Agave mckelveyana* | MT083850 |  |  |  |  |
| *Agave montium* | * |  |  |  |  |
| *Agave multifilifera* | * | * |  | * | * |
| *Agave nizandensis* | MT083851 | MT250114 | MT250152 |  | MT053333 |
| *Agave ocahui* | * |  |  |  |  |
| *Agave ovatifolia* | * |  |  |  |  |
| *Agave parryi* | MT083852 | MT250115 |  | MT250186 |  |
| *Agave parviflora* | AM884838.1 |  |  |  |  |
| *Agave sisalana* | MH768060.1 |  |  |  |  |
| *Agave peacockii* | * | * | * | * | * |
| *Agave pelona* | MT083853 | MT250116 | MT250153 | 250187 |  |
| *Agave pendula* | MT083854 |  |  |  |  |
| *Agave petrophila* | MT083855 | MT250117 | MT250154 |  |  |
| *Agave phillipsiana* | MT083856 |  |  |  |  |
| *Agave pintilla* | * | * | * | * | * |
| *Agave polianthiflora* | MT083857 | MT250118 | MT250155 |  | MT053334 |
| *Agave potatorum* | MT083858 | MT250119 | MT250156 |  | MT053335 |
| *Agave rhodacantha* | MT083859 | MT250120 | MT250157 | MT250188 | MT053336 |
| *Agave rzedowskiana* | MT083860 | MT250121 | MT250158 | MT250189 | MT053337 |
| *Agave salmiana* | MT083861 | MT250122 | MT250159 | MT250190 |  |
| *Agave scaposa* | MT083862 |  |  |  |  |
| *Agave schidigera* | MT083863 | MT250123 | MT250160 | MT250191 | MT053338 |
| *Agave schottii* | MT083864 |  |  |  |  |
| *Agave seemanniana* | MT083865 | MT250124 | MT250161 | MT250192 | MT053339 |
| *Agave shawii* | MT083866 | MT250125 | MT250162 |  |  |
| *Agave sobria* | MT083867 |  |  |  |  |
| *Agave striata* | MT083868 |  |  |  |  |
| *Agave tequilana* | MT083869 | MT250126 | MT250163 | MT250193 |  |
| *Agave titanota* | MT083870 | MT250127 | MT250164 |  | MT053340 |
| *Agave triangularis* | MT083871 | MT250128 | MT250165 | MT250194 | MT053341 |
| *Agave univittata* | * |  |  |  |  |
| *Agave victoriae-reginae* | MT083871 | MT250130 |  | MT250195 |  |
| *Agave vilmoriniana* | MT083872 |  |  |  |  |
| *Agave wocomahi* | MT083873 | MT250131 | MT250167 |  |  |
| *Agave zebra* | MT083874 | MT250132 | MT250168 |  | MT053342 |

* in submit process
